# The complete mitochondrial genome sequence of *Avena chinensis* (Fisch. ex Roem. & Schult.) Metzg., 1824 (Poaceae)

**DOI:** 10.1080/23802359.2026.2758976

**Published:** 2026-11-10

**Authors:** Dongfeng Shi, Xuchun Qin, Jing Li, Hongyu Wu

**Affiliations:** a Tianjin College, University of Science and Technology Beijing, Tianjin, PR China; b Inner Mongolia Minfeng Seed Industry Co., Ltd., Ulanqab, PR China

**Keywords:** *Avena chinensis*, mitochondrial genome, phylogenetic analysis, Poaceae

## Abstract

*Avena chinensis* is an oat species with potential agricultural value and genetic resources for oat improvement. Here, we report its first mitochondrial genome assembly, represented as a circular sequence of 549,864 bp with a GC content of 44.17%. The mitogenome contained 54 genes, including 34 protein-coding genes, 17 tRNA genes, and three rRNA genes. Phylogenetic analysis showed that *A. chinensis* was sister to *Avena longiglumis*, with *Poa pratensis* representing the closest sampled intergeneric relative. This study provides a valuable genomic resource for the further utilization and evolutionary research of *A. chinensis*.

## Introduction

Oat (*Avena* L.) is a widely cultivated cereal crop valued for both human consumption and animal feed. The genus *Avena* comprises approximately 30 species (Yan et al. 2016), including diploid, tetraploid, and hexaploid types (Ladizinsky 1998). Cultivated hexaploid oats possess 42 chromosomes, representing three distinct nuclear genomes (A, C, and D) (Loskutov and Rines 2011).

*Avena chinensis* (Fisch. ex Roem. & Schult.) Metzg. 1824, a cultivated hexaploid oat also known as oil oat or naked oat, is an annual herb (Li et al. 2019). Unlike hulled oats, its caryopses are hulless and separate easily from the husk at maturity. The absence of glumes simplifies processing and makes it more suitable for food production (Hackett 2018; Li et al. 2019). *A. chinensis* is mainly cultivated in northern and southwestern China, where it thrives in high-latitude, high-altitude, and arid or semi-arid environments due to its tolerance of cold, drought, and poor soils (Peltonen-Sainio et al. 2004). Its seeds are nutritionally rich, containing higher levels of protein and fat than many other cereals (Biel et al. 2009), and provide all eight essential amino acids (Biel et al. 2014). The seeds can be processed into traditional foods such as noodles, dumplings, and porridge, as well as modern products including breakfast cereals, biscuits, and bread. In addition, both seeds and straw are commonly used as livestock feed (Batalova et al. 2016).

Mitochondria are important organelles in plant cells that play central roles in cellular respiration, energy production, and multiple metabolic pathways (Plaxton and Podestá 2006; Van Aken 2021). The mitochondrial genome is also important for understanding plant genome organization, cytoplasmic inheritance, and evolutionary relationships (Chang et al. 2013; Chen et al. 2017). However, the mitochondrial genome of *A. chinensis* has not yet been reported. In this study, we sequenced and annotated the complete mitochondrial genome of *A. chinensis* and conducted phylogenetic analyses based on mitochondrial genomic data. These results provide valuable genomic resources and contribute to future studies on the evolution and genetic improvement of oat species.

## Materials and methods

The fresh leaves of *A. chinensis* were obtained from Tianjin, China (Geospatial coordinates: N 39°32′37″, E 117°23′41″). Pictures of the plant are shown in Figure 1. The plant specimen was identified by Dongfeng Shi (dongfshi@yeah.net). A specimen was deposited at the specimen collection of Tianjin College, University of Science and Technology Beijing, Tianjin, China (https://tj.ustb.edu.cn/ldxy/, contact person: Dongfeng Shi, dongfshi@yeah.net) under the voucher number TYMY-001 (Figure 1).

**Figure 1.**
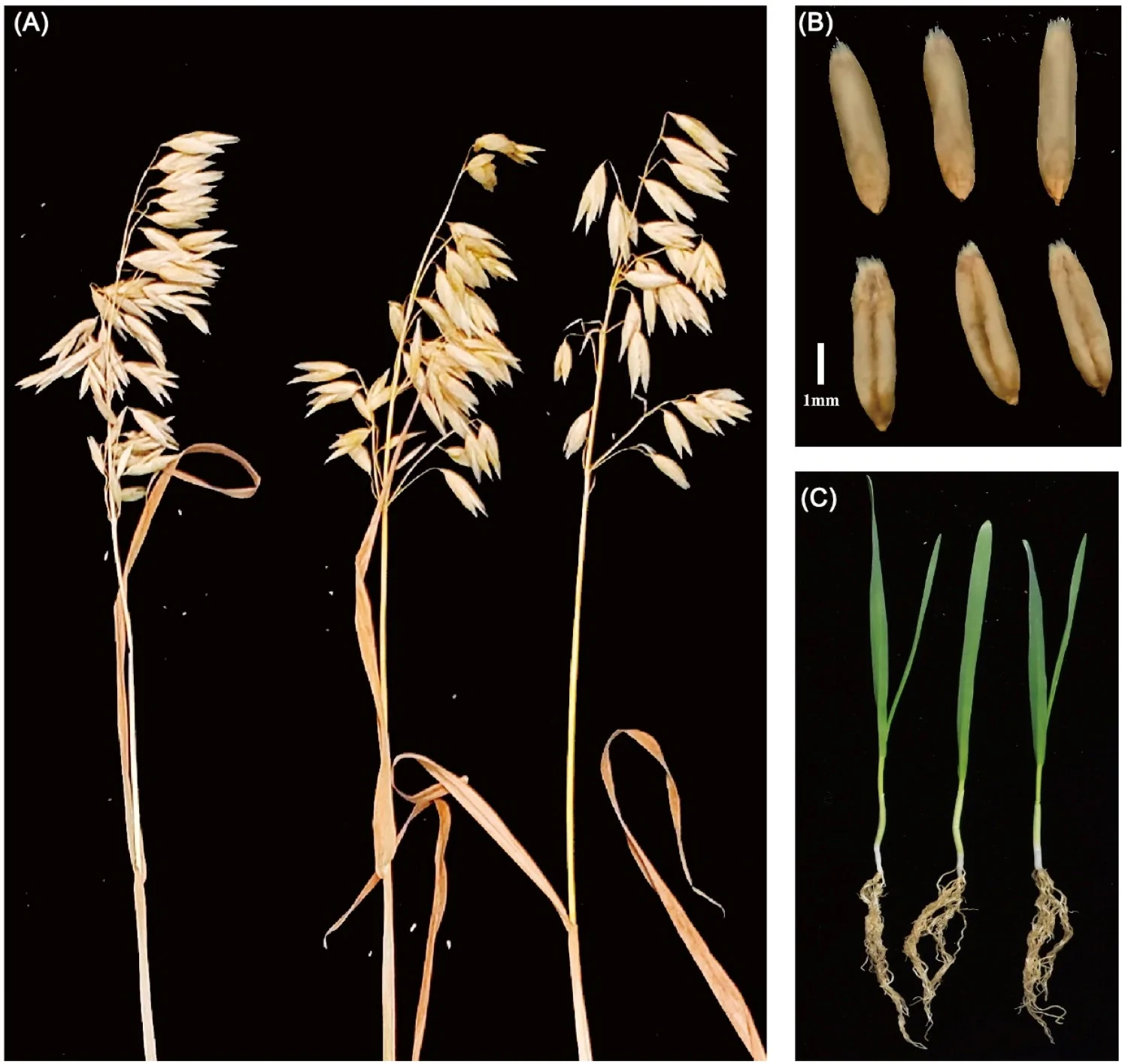
Morphological characteristics of *A. chinensis*, photographed by Dongfeng Shi (dongfshi@yeah.net) in Tianjin College, University of Science and Technology Beijing, Tianjin, China. (A) Mature panicle bearing caryopses, showing the open, lax branching pattern; (B) mature caryopses (grains), dorsal and ventral views, illustrating the oblong shape (approximately 4–5 mm in length) and pubescent lemma surfaces; the scale bar represents 1 mm; (C) seedling at the three-leaf stage, showing erect leaf blades and a well-developed root system.

Total genomic DNA was extracted using a Plant Genomic DNA Kit (Tiangen, Beijing, China) following the manufacturer’s instructions. Sequencing libraries were constructed for both Illumina and Oxford Nanopore platforms. A paired-end library with an insert size of approximately 350 bp was sequenced on the Illumina NovaSeq 6000 platform to generate 150 bp paired-end reads. A long-read library (∼10 kb) was sequenced on the PromethION platform (Oxford Nanopore Technologies, New York, NY). Illumina reads were processed using fastp with default parameters. Nanopore reads were filtered using NanoFilt, retaining reads with a mean quality score ≥15 and a minimum length of 1000 bp. Clean Nanopore and Illumina reads were then assembled using a hybrid strategy implemented in GSAT v1.12 (He et al. 2023). The assembly graph was examined with Bandage v0.8.1 (Wick et al. 2015). To assess assembly accuracy, Nanopore reads were mapped to the assembled contigs using minimap2 v2.29 (Li 2021). The mitochondrial genome was annotated using PMGA (Li et al. 2025), and tRNA annotations were confirmed using tRNAscan-SE (Chan et al. 2021). The circular genome map was generated with OGDRAW (Greiner et al. 2019). Cis-splicing and trans-splicing gene structures were visualized using PMGmap (Zhang et al. 2024). The annotated genome sequence has been deposited in GenBank under accession number PX851568.1.

For phylogenetic analysis, a total of 19 species from the family Poaceae, including *A. chinensis*, were selected. *Carex pseudochinensis* (PP465050.1) (Lee et al. 2025) from the family Cyperaceae was used as the outgroup. All mitochondrial genome sequences were obtained from the NCBI database. Shared protein-coding genes among the 20 species were extracted and concatenated using PhyloSuite v1.2.2 (Zhang et al. 2020). The concatenated sequences were aligned with MAFFT v7.505 (Katoh and Standley 2013). Phylogenetic reconstruction was performed using the maximum-likelihood (ML) method in IQ-TREE v3.0.1 (Lanfear et al. 2020) under the best-fit substitution model (TVM + F + R3). Branch support was evaluated using 1000 bootstrap replicates. The phylogenetic tree was visualized using the Interactive Tree of Life (iTOL) online platform (Letunic and Bork 2024).

## Results

After quality filtering, 40.01 Gb of Illumina reads and 30.77 Gb of Nanopore reads were obtained. The complete circular mitochondrial genome of *A. chinensis* is 549,864 bp in length, with an average sequencing depth of 76.12× (Figure S1). The base composition is 28.00% A, 27.84% T, 22.18% G, and 21.98% C, with an overall GC content of 44.17%. A total of 67 gene copies were annotated, representing 54 unique genes, including 34 protein-coding genes, 17 tRNA genes, and three rRNA genes (Figure 2). The 34 protein-coding genes were classified into nine functional categories: ATP synthase (five genes: *atp1*, *atp4*, *atp6*, *atp8*, *atp9*), cytochrome c biogenesis (four genes: *ccmB*, *ccmC*, *ccmFC*, *ccmFN*), ubiquinol cytochrome c reductase (one gene: *cob*), cytochrome c oxidase (three genes: *cox1*, *cox2*, *cox3*), maturase (one gene: *matR*), membrane transport protein (one gene, *mttB*, present in two copies), NADH dehydrogenase (nine genes: *nad1*, *nad2*, *nad3*, *nad4*, *nad4L*, *nad5*, *nad6*, *nad7*, *nad9*), large subunit ribosomal proteins (two genes: *rpl16* and *rpl5*), and small subunit ribosomal proteins (eight genes: *rps1*, *rps2*, *rps3*, *rps4*, *rps7*, *rps12*, *rps13*, *rps14*). Eight genes containing introns were identified. Among them, three cis-splicing genes (*ccmFC*, *cox2*, and *rps3*) each harbor one intron (Figure S3). Four trans-splicing genes (*nad1*, *nad2*, *nad5*, and *nad7*) each contain four introns, and *nad4* contains three introns (Figure S4).

**Figure 2.**
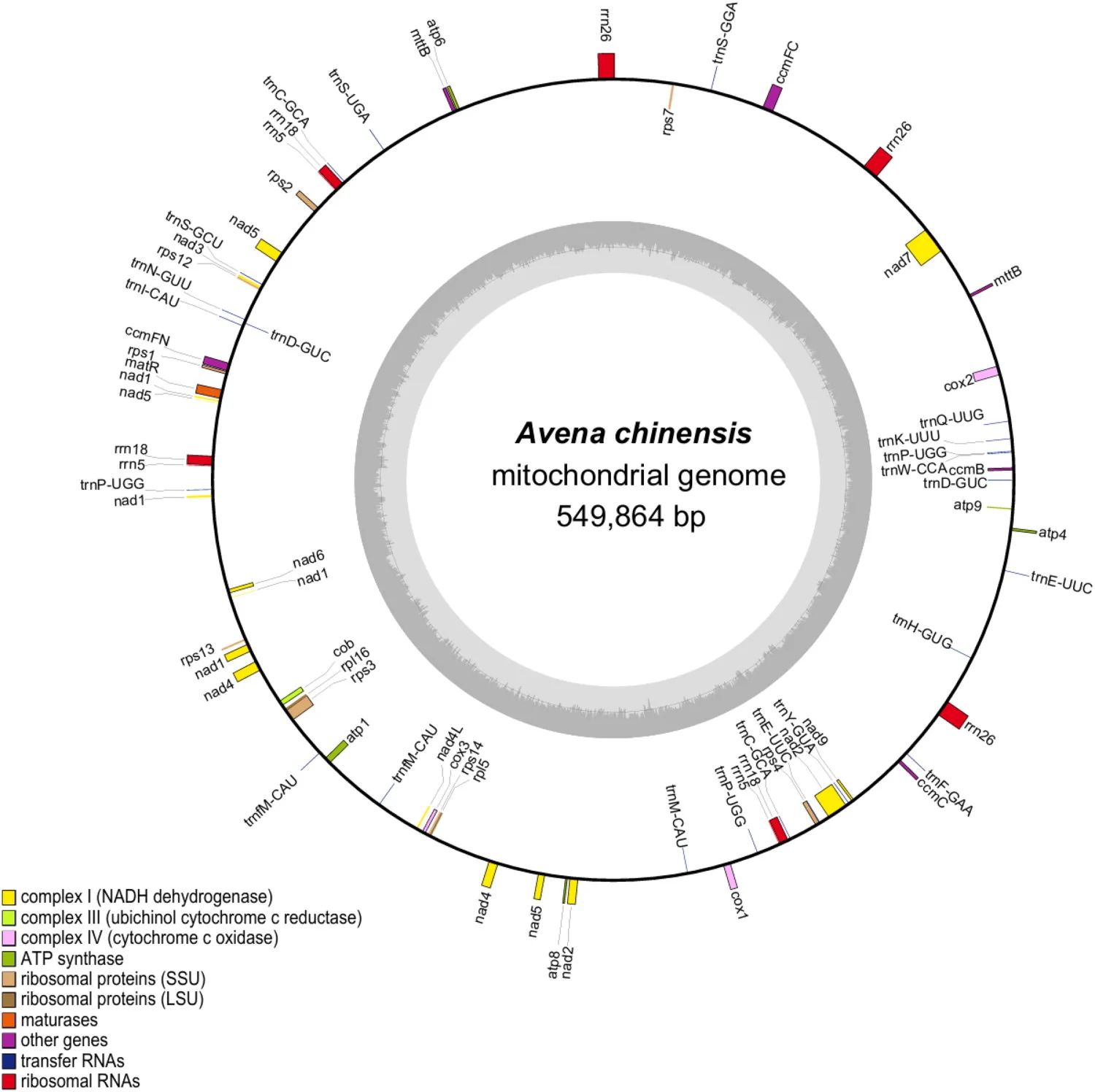
Circular map of the *A. chinensis* mitochondrial genome, showing 54 annotated genes grouped by function and color-coded according to their functional categories. Features on the clockwise and counter-clockwise strands are displayed on the inner and outer circles, respectively.

A total of 14 shared protein-coding genes (*atp9*, *ccmC*, *cox1*, *cox2*, *cob*, *nad1*, *nad2*, *nad3*, *nad5*, *nad6*, *nad7*, *rps3*, *rps7*, and *rps12*) were extracted and used for phylogenetic reconstruction. The resulting tree clearly resolved the evolutionary relationships among the sampled species. *A. chinensis* showed a close phylogenetic relationship with *Avena longiglumis* (OQ450323.1) within the genus *Avena* (Figure 3). At the intergeneric level, it was more closely related to *Poa pratensis* (OQ612620.1) among the examined taxa. Overall, the phylogenetic tree was well supported and accurately reflected the evolutionary relationships among these species.

**Figure 3.**
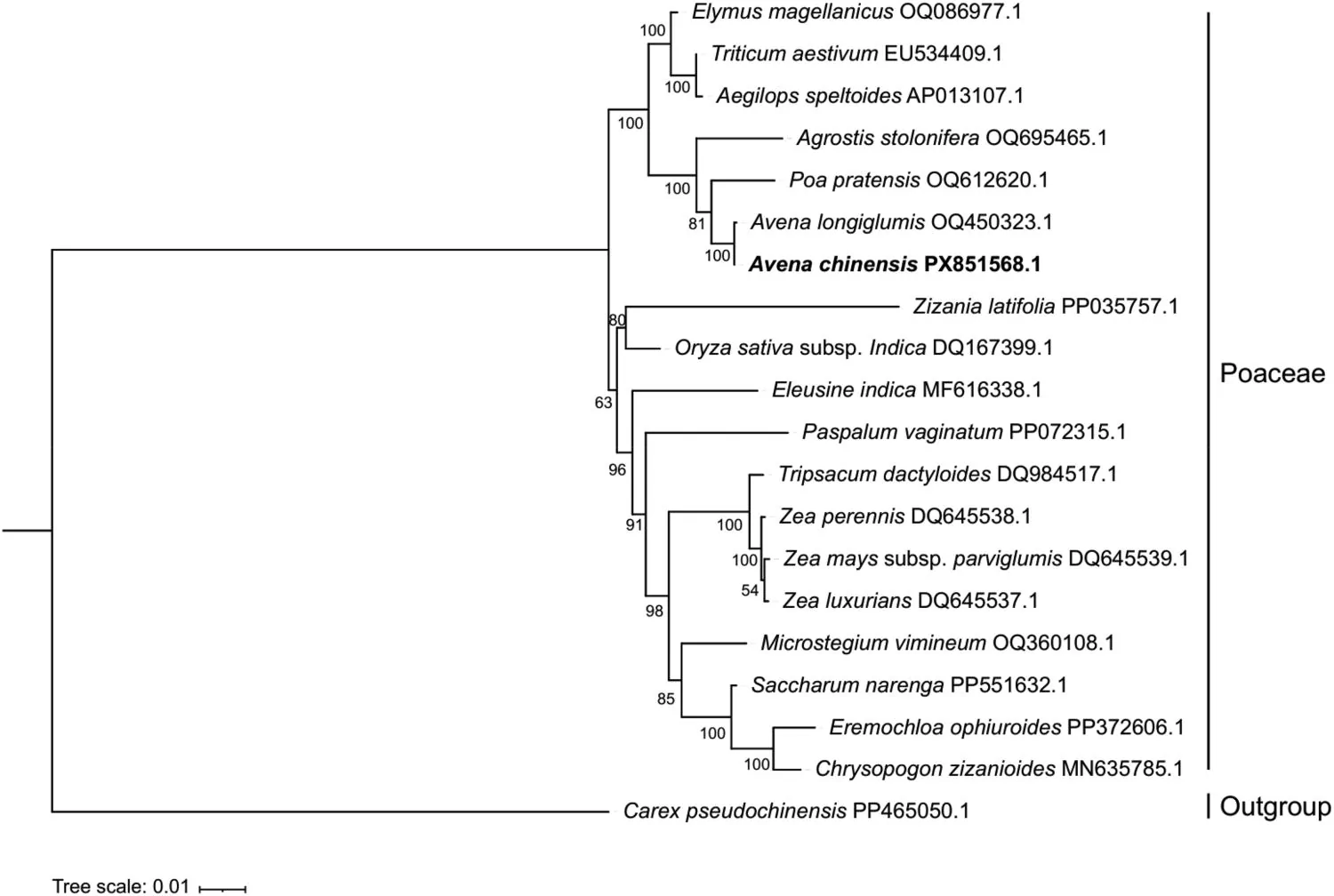
The phylogenetic relationships of *A. chinensis* with 19 other plant species were inferred based on mitochondrial genome data. A ML tree was constructed using the shared protein-coding genes among these species. Bootstrap support values are indicated at each node. *A. chinensis* is highlighted in bold. The following species were included in the analysis: *Elymus magellanicus* (OQ086977.1) (Chen et al. 2023), *Triticum aestivum* (EU534409.1) (Cui et al. 2009), *Aegilops speltoides* (AP013107.1), *Agrostis stolonifera* (OQ695465.1) (Li et al. 2023), *Poa pratensis* (OQ612620.1), *Avena longiglumis* (OQ450323.1) (Liu et al. 2023), *Zizania latifolia* (PP035757.1) (Luo et al. 2024), *Oryza sativa* subsp. *indica* (DQ167399.1), *Eleusine indica* (MF616338.1), *Paspalum vaginatum* (PP072315.1), *Tripsacum dactyloides* (DQ984517.1), *Zea perennis* (DQ645538.1), *Zea mays* subsp. *parviglumis* (DQ645539.1), *Zea luxurians* (DQ645537.1), *Microstegium vimineum* (OQ360108.1), *Saccharum narenga* (PP551632.1), *Eremochloa ophiuroides* (PP372606.1) (Zhao et al. 2025), and *Chrysopogon zizanioides* (MN635785.1), with *Carex pseudochinensis* (PP465050.1) (Lee et al. 2025) as the outgroup.

## Discussion and conclusions

Plant mitochondrial genomes are known for frequent recombination and rearrangements, intracellular gene transfer, and repetitive sequences (Gualberto and Newton 2017). These features result in great structural diversity. Genome configurations can range from a single circular molecule to complex mixtures of circular and linear forms. Similar repeat-mediated recombination events have been reported in other plant species, including *Ventilago leiocarpa* (Guo et al. 2024), *Melia azedarach* (Hao et al. 2024), and *Brasenia schreberi* (Shan et al. 2024). In this study, we visualized the long-read assembly graph using Bandage v0.8.1. The graph showed a circular configuration of the mitochondrial genome (Figure S2). Similar configurations have been reported in *Psophocarpus tetragonolobus* (Singh et al. 2025), *Glycyrrhiza glabra* (Zhou et al. 2025), and *Brassica rapa* var. *Purpuraria* (Gong et al. 2024). However, repeat-mediated subgenomic conformations cannot be entirely ruled out. Further repeat-spanning read pair analysis is needed to assess their presence.

Genome annotation identified all expected genes with no apparent gene loss, indicating that the assembly is reliable. During annotation, several non-canonical codons were identified. Specifically, *nad1* and *nad4L* used ACG as the start codon, *rpl16* used GTG as the start codon, and *ccmFC* used CGA as the stop codon. Such non-canonical codons have also been found in *Prunus salicina* (Fang et al. 2021), *Viburnum chinshanense* (Zhu et al. 2023), and *Hemerocallis citrina* (Zhang et al. 2022). Based on previous reports, RNA editing may explain some of these non-canonical codons; however, this interpretation remains speculative, as it has not been experimentally validated in this species.

To date, only one complete mitochondrial genome has been reported in the genus *Avena*, that of *A. longiglumis* (OQ450323.1). Its genome size is 548,445 bp, which is comparable to that of *A. chinensis*. In this study, we present the first complete mitochondrial genome of *A. chinensis*. Phylogenetic analysis supported a sister relationship between *A. chinensis* and *A. longiglumis*. However, the limited sampling of *Cyperaceae* as an outgroup may affect the resolution of deeper phylogenetic relationships within Poaceae. This genome provides valuable molecular data for future phylogenetic and evolutionary studies within Poaceae.

## Supplementary Material

Supplemental Material

## Data Availability

The raw sequencing data generated from the Illumina and Nanopore platforms have been deposited in NCBI. The associated BioProject, BioSample, and SRA accession numbers are PRJNA1401544, SAMN54566665, SRR36783273 (Illumina), and SRR36783274 (Nanopore). The complete mitochondrial genome sequence has been deposited in the NCBI GenBank database under accession number PX851568.1.
