# Supplementary material for: The complete mitochondrial genome sequence of Avena chinensis (Fisch. ex Roem. & Schult.) Metzg., 1824 (Poaceae)
Source: Mitochondrial DNA B Resour. 2026 Nov 10;11(12):1405–10. doi: 10.1080/23802359.2026.2758976 (PMC13757913; doi:10.1080/23802359.2026.2758976)
Supplement: Supplementary file 1 [file TMDN_A_2758976_SM5055.docx]

Figure S1. Coverage profile and sequencing depth across the mitochondrial genome assembly of *A. chinensis*. The x-axis shows genomic coordinates, and the y-axis represents sequencing depth per nucleotide position.

Figure S2. The assembly graph of the mitochondrial gensome of *A. chinensis* visualized using Bandage shows a single circular contig, ctg1 (549,864 bp).

Figure S3. Structural organization of cis-splicing genes containing more than two exons in the mitochondrial genome of *A. chinensis*. For each gene, the schematic consists of three layers. The top layer shows the gene name. The middle layer represents the genomic sequence context, with colored boxes indicating exons and numbers along the sequence denoting annotated exon boundaries based on genome annotation; connecting lines indicate the relative positions of these sites. The bottom layer illustrates the mature mRNA structure after splicing, showing transcriptional orientation (5′–3′) and exon lengths in base pairs.

Figure S4. Structural organization of trans-splicing genes in the mitochondrial genome of *A. chinensis*. For each gene, the schematic consists of three layers. The top layer shows the gene name. The middle layer represents the genomic sequence context, with colored boxes indicating exons distributed across separate genomic regions and numbers along the sequence denoting annotated exon boundaries based on genome annotation; connecting lines indicate the relative positions of these sites. The bottom layer illustrates the mature mRNA structure after trans-splicing, showing transcriptional orientation (5′–3′) and exon lengths in base pairs.
